# Safe Limits of Contrast Media for Contrast-Induced Nephropathy: A Multicenter Prospective Cohort Study

**DOI:** 10.3389/fmed.2021.701062

**Published:** 2021-08-20

**Authors:** Zhiqiang Nie, Yong Liu, Chao Wang, Guoli Sun, Guo Chen, Zuxun Lu

**Affiliations:** ^1^Department of Social Medicine and Health Management, School of Public Health, Tongji Medical College, Huazhong University of Science and Technology, Wuhan, China; ^2^Department of Epidemiology, Guangdong Provincial Key Laboratory of Coronary Heart Disease Prevention, Guangdong Cardiovascular Institute, Guangdong Provincial People's Hospital, Guangdong Academy of Medical Sciences, Guangzhou, China; ^3^Department of Cardiology, Guangdong Provincial Key Laboratory of Coronary Heart Disease Prevention, Guangdong Cardiovascular Institute, Guangdong Provincial People's Hospital, Guangdong Academy of Medical Sciences, Guangzhou, China

**Keywords:** contrast-induced nephropathy, coronary angiography, percutaneous coronary intervention, glomerular filtration rate, safe limits

## Abstract

**Background:** The safe level of contrast media volume (CV) is an important modifiable risk factor for contrast-induced nephropathy (CIN). The safe limit of CV remains unclear and is limited to single-center studies. Our objective was to determine the association between the ratio of contrast volume-to-glomerular filtration (CV/GFR) and CIN in patients undergoing coronary angiography (CAG) or percutaneous coronary intervention (PCI).

**Methods:** We assessed the association between CV/GFR and the risk of CIN in 4,254 patients undergoing CAG or PCI from the year 2013 to 2016 and enrolled in the REICIN (REduction of rIsk for Contrast-Induced Nephropathy), a prospective, multicenter, observational cohort study. CV/GFR was calculated at the five primary GFR equation.

**Results:** Sixty-nine (1.7%) patients with a median contrast volume-to-chronic kidney disease epidemiology collaboration (CV/CKD-EPI) ratio of 2.16 (1.30–3.93) have suffered from CIN. The CV/CKD-EPI demonstrated the best performance of model fit, discrimination (area under curve = 0.736), calibration, reclassification, and equation conciseness (1 variable). The CV/CKD-EPI ≥1.78 was the statistical significance associated with CIN [adjusted odds ratio, 4.64 (2.84–7.56); *p* < 0.001]. Furthermore, similar results were found in the subgroup analyses.

**Conclusions:** The CV/CKD-EPI showed the best performance in patients undergoing CAG or PCI. CV/CKD-EPI ≥1.78 could be a more reliable and convenient predictor of CIN. Intraprocedural preventive measures should include a priori calculation of CV/GFR to limit contrast volume.

## Introduction

Contrast-induced nephropathy (CIN) is a common but serious complication of coronary angiography (CAG) and/or percutaneous coronary intervention (PCI). CIN is defined as a decline in kidney function that occurred in a narrow time window after administration of iodinated contrast agent (1). Although several factors have been identified as risk factors of CIN, such as chronic kidney disease (CKD), diabetes mellitus, hemodynamic instability, gender, and age, they are not typically modifiable. Recently, the importance of modifiable influencing factors of CIN, including the safe level of contrast media volume (CV) has been increasingly recognized to minimize the nephrotoxicity (2).

Contrast media is mainly excreted *via* kidneys pharmacokinetically. Several previous studies have investigated the safe level of CV for CIN after CAG or PCI using a single pharmacokinetic index, such as contrast volume-to-creatinine clearance (CV/CrCl) or contrast volume-to-glomerular filtration rate (CV/GFR) (3–10). Smaller CV/GFR cutoffs, corresponding to lower levels of CV, has been used to facilitate clinical decision. According to the European Society of Cardiology/European Association for Cardio-Thoracic Surgery (ESC/EACTS) guidelines (11), the recommended cutoff value of CV/GFR was 3.7, and a CV/GFR value >3.7 increases the risk of CIN (within 24 h) significantly (3, 12). But in real clinical practices, when the ratio is <3.7, a significant number of patients still develop CIN. Another U.S. cohort study has demonstrated that CV/GFR >3 dramatically elevated the risk of CIN (13), meanwhile, further cutoff should be optimized in the range of CV/GFR <3. Our previous study has suggested that CV/GFR >2.62 was a significant and independent predictor of CIN (within 72 h), but these data were collected in a single center (7). Thus far, there has been no prospective cohort study with multicenter recruitment to validate the cutoff value of CV/GFR (14, 15). On the other hand, since the existing recommended CV/GFR cutoffs were determined based on Canadian and American populations, whether these values were also appropriate for Chinese patients remain controversial.

Meanwhile, there are 10 algorithms to calculate GFR (Supplementary Table 1), such as Cockcroft–Gault (C-G) (16), modification of diet in renal disease (MDRD) (13, 17), chronic kidney disease epidemiology collaboration (CKD-EPI) (18), etc. They were developed based on different populations. For instance, C-G was derived from natural population, while MDRD and CKD-EPI were derived from CKD population. Previous studies show conflicting results because of different study populations, different gold standard GFR measurements, and different creatinine assay calibration (19, 20). However, there is no study that assessed the utility of all the 10 algorithms in CAG or PCI patients. Evidence-based recommendations considering multiple estimated GFR (eGFR) algorithm to guide the best CV/GFR strategies for CAG or PCI patients are still lacking. Therefore, we aimed to determine the optimal CV/GFR equation in predicting CIN and to define the safe dose of contrast media on the basis of GFR in this prospective study.

## Methods

### Study Population

The REduction of rIsk for Contrast-Induced Nephropathy (REICIN) study (trial registration: ClinicalTrials.govNCT01402232) is a prospective, multicenter, observational cohort study that recruited patients referred for CAG or PCI in 12 hospitals in Guangdong, Fujian, and Xinjiang, China, from January 2013 to February 2016 (follow-up is ongoing). Details of the site investigators and hospitals are provided in Supplementary Table 2. Details of the study procedure and inclusion and exclusion criteria are mentioned in Supplementary Figure 1. The study protocol was approved by the institutional Ethics Research Committee of Guangdong General Hospital (no. GDREC2012141H). All patients gave written informed consent before participating in this study.

### Patient Management and Data Collection

The selection of contrast media was at the discretion of the operating physician within the dictates of the individual hospital policy. CV was expressed only in terms of volume in milliliters in this study because the CM concentration used during coronary procedures usually varies within narrow ranges, i.e., 320–370 mg I/ml as in the previous study (3, 13). CAG was performed according to standard clinical practice, using standard guide catheters, guidewires, balloon catheters, and stents *via* the femoral or radial approach. The most recent preoperative serum creatinine level and other laboratory biomarkers were defined as the baseline value. Measurements were repeated after CAG or PCI on the first, second, and third postoperative days. The beginning and end time of CAG or PCI were recorded. PCI techniques were selected at the discretion of the interventional cardiologist. We also collected the demographic data of patients and procedural characteristics from original records and hospitals' electronic medical records.

### Study Endpoints and CV/GFR

The primary outcome of this analysis was CIN, which was defined as an impairment in renal function resulting in ≥0.5 mg/dl absolute increase in serum creatinine from baseline within 24–48 h. For each patient, we, respectively, estimated volume-to-GFR with five equations: contrast volume-to-chronic kidney disease epidemiology collaboration (CV/CKD-EPI) (18, 19, 21), contrast volume–to–Cockcroft–Gault (CV/C-G) (16), contrast volume-to-full age spectrum (CV/FAS) (22), contrast volume-to-modification of diet in renal disease study (CV/MDRD) (23, 24), and contrast volume-to-abbreviated modification of diet in renal disease study (CV/aMDRD) (25) (Supplementary Table 1). Because C-G has been conventionally used for evaluating the renal dosing (26) and CKD-EPI was established on minimalist clinical measurements, these two results were preferentially reported. The Berlin Initiative Study 1 (BIS1) and revised Lund-Malmö (LM-rev) algorithms were not included in the primary analysis because of their poor predictive performance [area under the receiver operating characteristics curve (AUC) <0.70], and BIS1 is only applicable for the elderly. The isotope dilution mass spectrometry (MDRD-IDMS) algorithm was not included because GFR was measured by the Roche enzymatic method but not IDMS in this algorithm. MDRD7-cn and aMDRD-cn were not analyzed since they were same algorithms with different coefficients, thus exhibiting the same predictive performance. Because of these limitations, we rule out the five algorithms of GFR equation.

### Statistical Analyses

Missing data were imputed using the multivariate imputations by chained equations method with missing-at-random assumptions (Supplementary Table 3). Five copies of the data, each with missing values imputed, estimates of the parameters of interest were averaged across the copies. All results shown are the results after the multiple imputations of data (Table 1).

**Table 1:** Baseline characteristics of patients who developed CIN.

**Risk factor**	**CIN**,	**No CIN**,	***p*-value^†^**
	***n* (%)**	***n* (%)**	
**Total** ^*^	69 (1.7)	4,185 (98.3)	
**Demographic**			
Age, years	71 (62–76)	63 (55–71)	<0.001
≥80	9 (13.0)	211 (5.0)	
60–79	46 (66.7)	2,383 (56.9)	
<60	14 (20.3)	1,591 (38.0)	
Male	49 (71.0)	3,103 (74.1)	0.556
Weight, kg	65 (58–69)	65 (58–71)	0.686
BMI	24 (22–26)	24 (22–26)	0.741
History of smoking	25 (36.2)	1,563 (37.3)	0.849
**Medical history**			
Diabetes mellitus	24 (34.8)	1,132 (27.0)	0.152
Previous CABG	0 (0.0)	7 (0.2)	0.734
Hyperlipidemia	9 (13.0)	506 (12.1)	0.81
Anemia	30 (43.5)	1,206 (28.8)	0.008
Previous MI	5 (7.2)	389 (9.3)	0.560
PVD	0 (0.0)	6 (0.1)	0.753
Anterior infarction	9 (13.0)	381 (9.1)	0.261
Cardiogenic shock	10 (14.5)	28 (0.7)	<0.001
CHF	30 (43.5)	911 (21.8)	<0.001
CVD	5 (7.2)	177 (4.2)	0.219
HF	30 (43.5)	803 (19.2)	<0.001
Hypoalbuminemia	10 (14.5)	190 (4.5)	<0.001
Stroke	5 (7.2)	177 (4.2)	0.219
**Clinical conditions**			
Presence of ACS	37 (53.6)	1,953 (46.7)	0.251
UA/NSTEMI	28 (40.6)	1,738 (41.5)	0.874
Anterior STEMI	23 (33.3)	979 (23.4)	0.054
Cardiac arrest	0 (0.0)	7 (0.2)	0.734
Peri-hypotension	8 (11.6)	116 (2.8)	<0.001
Peri-IABP	8 (11.6)	72 (1.7)	<0.001
LVEF <40%	22 (31.9)	339 (8.1)	<0.001
NYHA class level ≥3	3 (4.3)	292 (7.0)	0.634
Killip class level ≥3	6 (8.7)	175 (4.2)	0.147
**Laboratory measurements**			
Preprocedural plasma glucose	7 (6–10)	6 (5–8)	0.001
Min of hemoglobin	108 (93–133)	132 (120–143)	<0.001
Hct	38 (35–42)	40 (37–43)	0.005
BUN	7 (5–10)	5 (4–6)	<0.001
HDL-C, mmol/L	1 (1–1)	1 (1–1)	0.635
ALB-C, mmol/L	34 (32–37)	37 (35–40)	<0.001
CK	165 (81–1,024)	97 (66–164)	0.001
CK-MB	17 (9–84)	10 (7–16)	<0.001
**Procedure**			
Multivessel stent	11 (15.9)	659 (15.7)	0.965
Diseased vessel ≥1	60 (87.0)	3,360 (80.3)	0.166
Diseased multivessel	50 (72.5)	2,382 (56.9)	0.01
CTO	16 (23.2)	712 (17.0)	0.177
PCI	52 (75.4)	2,509 (60.0)	0.009
Number of stents	1 (0–2)	1 (0–2)	0.039
Emergent PCI	14 (20.3)	492 (11.8)	0.030
Mehran integer score	9 (6–13)	4 (1–7)	<0.001
Exceeding MACD	8 (11.6)	52 (1.2)	<0.001
CV/GRF			
CV/CKD-EPI	2.16 (1.30–3.93)	1.15 (0.66–1.79)	<0.001
CV/C-G	2.50 (1.45–4.39)	1.27 (0.74–2.01)	<0.001
CV/FAS	2.32 (1.42–3.59)	1.21 (0.70–1.89)	<0.001
CV/MDRD	2.33 (1.44–4.05)	1.25 (0.72–1.96)	<0.001
CV/aMDRD	2.52 (1.46–4.13)	1.33 (0.71–2.11)	<0.001

*Categorical data are presented as number (%), and continuous data are presented as median (interquartile range)*.

* *Data include imputed data for those with missing values*.

† *Comparison of complete cases group and patients with at least one missing value group*.

*ACEI/ARB, angiotensin converting enzyme inhibitor/angiotensin receptor blocker; ACS, acute coronary syndrome; ALB, albumin; ARF, acute renal failure; BMI, body mass index; BP, blood pressure; BUN, blood urea nitrogen; CABG, coronary artery bypass graft; CCB, calcium channel blocker; CHF, congestive heart failure; CIN, contrast-induced nephropathy; CK, creatine kinase; CK-MB, creatine kinase-muscle/brain; CTO, chronic total occlusion; CVD, cardiovascular disease; eGFR, estimated glomerular filtration rate; Hct, hematocrit; HDL-C, high-density lipoprotein cholesterol; HF, heart failure; HR, heart rate; IABP, intra-aortic balloon pump; LVEF, left ventricular ejection fraction; MI, acute myocardial infarction; NSTEMI, non-ST-elevation myocardial infarction; NYHA, New York Heart Association; PCI, percutaneous coronary intervention; PVD, peripheral vascular disease; Scr, serum creatinine; UA, unstable angina*.

**Figure 1:**
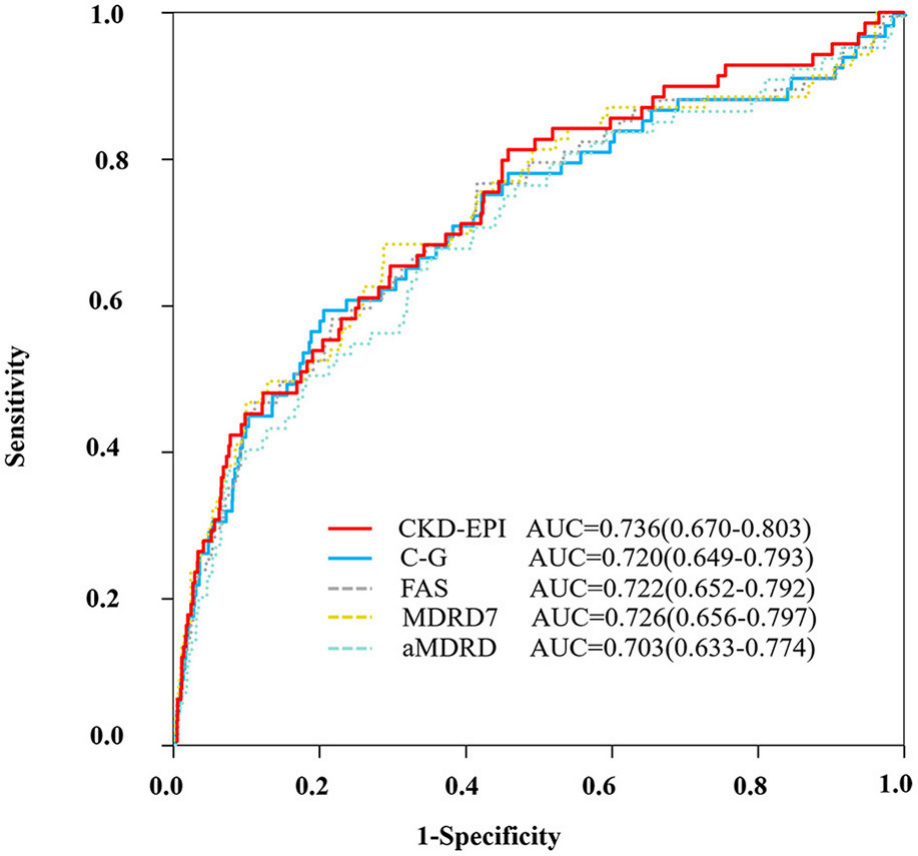
ROC curve. ROC curve to evaluate the diagnostic performance of the ratio of CV/GFR to predict CIN according to the different equations (CKD-EPI, C-G, FAS, MDRD7, and aMDRD). aMDRD, abbreviated modification of diet in renal disease; AUC, area under the receiver operating characteristics curve; C-G, cockcroft–gault; CKD-EPI, chronic kidney disease epidemiology collaboration; FAS, full age spectrum; MDRD, modification of diet in renal disease study.

**Figure 2:**
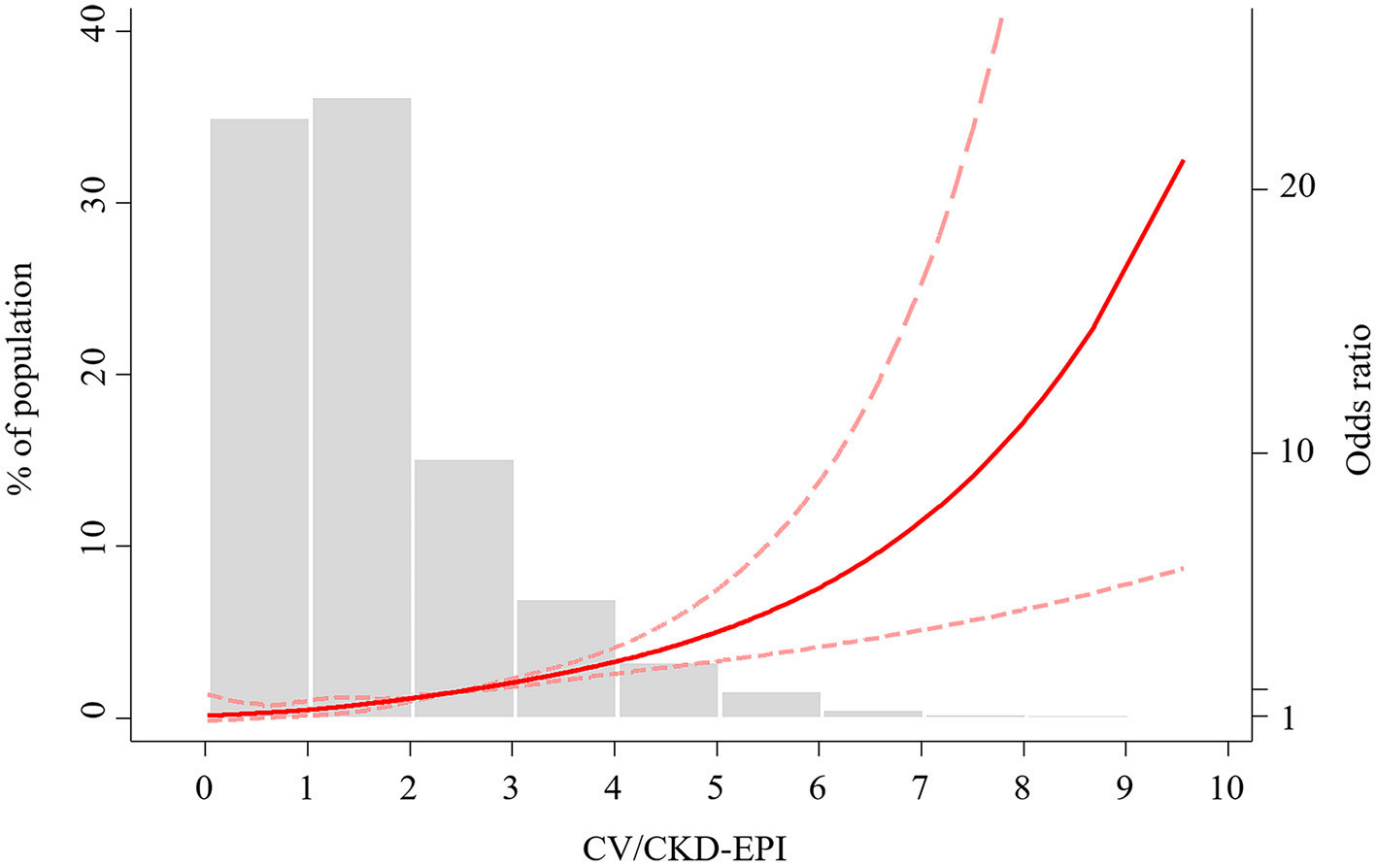
Restricted cubic spline of CV/CKD-EPI ratio and CIN. CV/CKD-EPI, contrast volume to chronic kidney disease epidemiology collaboration; CIN, contrast-induced nephropathy; OR, odds ratio.

**Figure 3:**
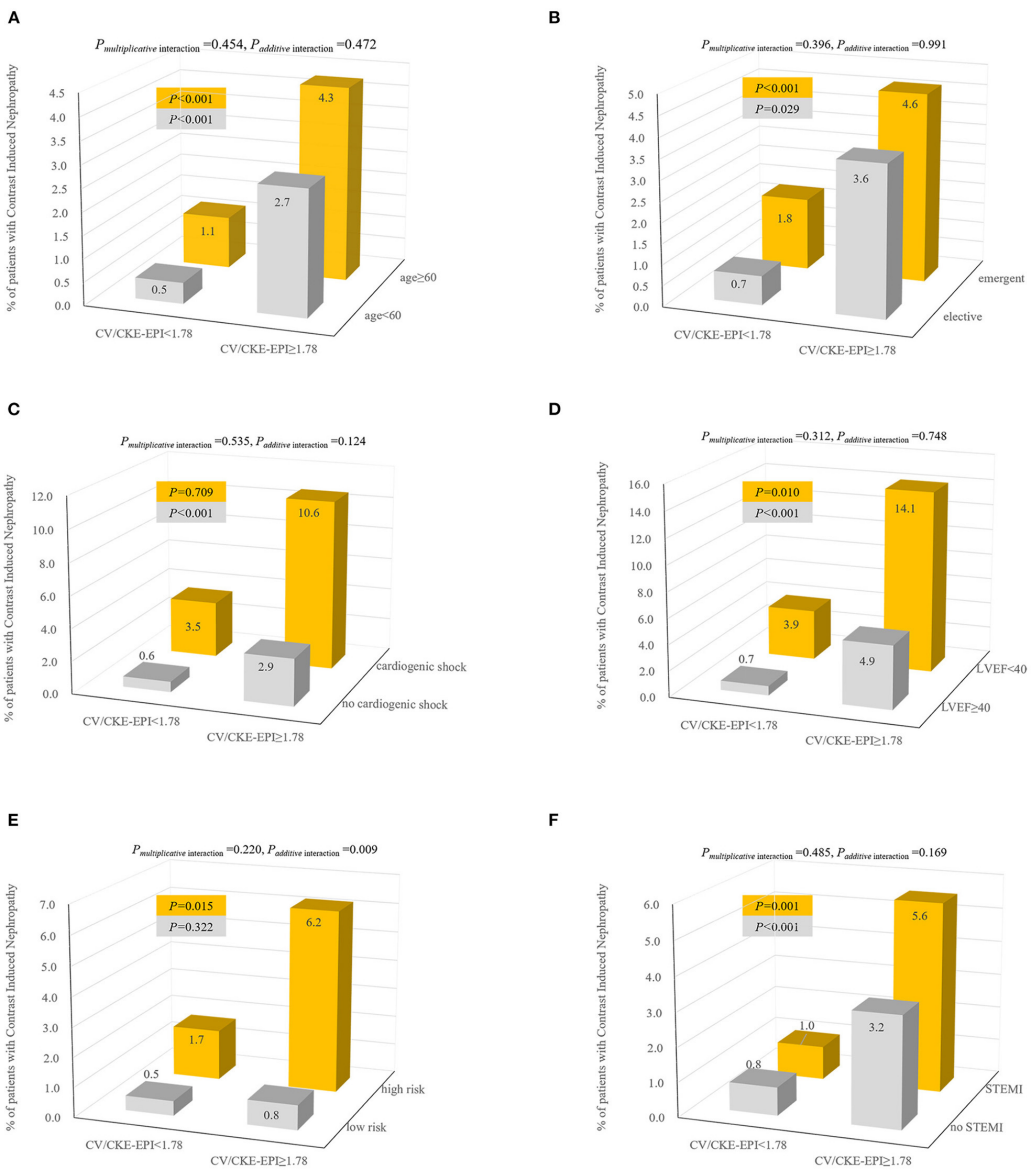
Incidence and interaction of CIN according to joint distribution of CV/CKD-EPI cutoff 1.78 and patients with elder age **(A)**, elective PCI **(B)**, cardiogenic shock **(C)**, LVEF **(D)**, high-risk level of Mehran score **(E)**, and STEMI **(F)**. Interaction effects were calculated by multivariate model adjusted for covariates (forward stepwise method): age, anemia, cardiogenic shock, CHF, HF, hypoalbuminemia, peri-hypotension, peri-IABP, LVEF <40%, preprocedural plasma glucose, min of hemoglobin, Hct, BUN, ALB, CK, CK-MB, diseased multivessel, PCI, number of stents, emergent PCI, Mehran integer score, and exceeding MACD. CV/CKD-EPI, contrast volume to chronic kidney disease epidemiology collaboration; CIN, contrast-induced nephropathy; LVEF, left ventricular ejection fraction; STEMI, segment elevated myocardial infarction.

Continuous variables are expressed as mean ± standard deviation, and discrete variables are described as frequency counts and percentages. The differences in continuous variables were analyzed with the *t*-test and Wilcoxon test as needed. Discrete variables between groups were evaluated by the Chi-square test and Fisher's exact test. We estimated empirical AUC for comparing CV/C-G, CV/CKD-EPI, CV/FAS, CV/MDRD7, and CV/aMDRD with bootstrap method (1,000 resamples). Receiver operator characteristic (ROC) curve analysis was used to determine the optimal cut-point for CV/GFR in this population and compared AUC with the DeLong and Clarke-Pearson methods (Figure 1). Additionally, we modeled CV/CKD-EPI as restricted quadratic splines with knots at the 5, 50, and 95th percentiles of its distribution to provide a smooth and flexible description of the dose–response relationship between CV/CKD-EPI and CIN (Figure 2). Risk factors were initially screened for univariate association with CV/CKD-EPI, and external multivariable logistics regression (according to non-significant multilevel effect on collaboration centers with an intraclass correlation coefficient of 0.005; data not shown) adjusted for other important baseline characteristics was identified in a forward stepwise manner using a *p*-value criterion of <0.05 (Table 1). The optimal threshold was determined using an ROC curve analysis following Youden's index. AUC was evaluated for discrimination, and Hosmer–Lemeshow (H-L) statistic, Akaike information criteria (AIC), the Brier score were applied to compare the calibration. The goodness of reclassification was evaluated by integrated discrimination improvement (IDI), and the category net reclassification index (NRI) was calculated (Table 2). We also tested the joint association between CV/CKD-EPI cutoffs according to the joint distribution of subgroups (Figure 3): elderly adult, patients for segment elevated myocardial infarction (STEMI), emergent PCI, patients with cardiogenic shock, left ventricular ejection fraction (LVEF) <40, and high risk level of Mehran score. Measures of interaction for the primary outcome are presented on multiplicative scales and multiplicative scale (27, 28). Interaction contrast ratio with 95% confidence interval (CI) was used to evaluate additive interaction. All analyses were performed using SAS software v9.4 (SAS, Cary, North Carolina) and R v3.6.0 (R Foundation for Statistical Computing, Vienna, Austria).

**Table 2:** Comparison of the five equations of CIN using IDI and NRI.

**Equation definition**	AUC 95%CI^*^	***P_delong_***		**AIC**	**Brier score**	**IDI 95%CI**	***P_***IDI***_***	**NRI 95%CI**	***P_***NRI***_***	**Variable^†^**
CV/CKD-EPI	0.736 (0.670–0.803)	Refrence	0.142	640.792	0.015	Refrence	Refrence	Refrence	Refrence	1
CV/C-G	0.720 (0.649–0.793)	0.142	Refrence	652.730	0.016	0.012 (−0.124 to 0.148)	0.864	−0.162 (−0.393 to 0.067)	0.179	2
CV/FAS	0.722 (0.652–0.792)	0.020	0.807	651.084	0.016	−0.141 (−0.218 to 0.065)	<0.001	−0.487 (−0.725 to −0.250)	<0.001	1
CV/MDRD	0.726 (0.656–0.797)	0.148	0.583	643.480	0.015	0.069 (0.005 to 0.133)	0.003	−0.096 (−0.287 to 0.094)	0.426	3
CV/aMDRD	0.703 (0.633–0.774)	0.010	0.286	663.919	0.016	−0.087 (−0.321 to 0.145)	0.461	−0.140 (−0.364 to 0.083)	0.247	1

*a-MDRD, abbreviated Modification of Diet in Renal Disease; AUC, area under the receiver operating characteristics curve; CI, confidence interval; C-G, CockcroftGault; CKD-EPI, Chronic Kidney Disease Epidemiology Collaboration; eGFR, estimating glomerular filtration rate; FAS, Full Age Spectrum; IDI, integrated discrimination improvement; IQR, interquartile range; MDRD, Modification of Diet in Renal Disease Study; NRI, net reclassification index*.

* *A bootstrap method (1,000 resamples) provided estimates of 95% CIs*.

† *External variables besides gender and age*.

### Sample Size Consideration

The *post hoc* sample size was calculated according to the rule of thumb of Vittinghoff et al., Peduzzi, and Harrell et al. (29–31), namely, the number of events per variable (EPV) of 5 to 10 or greater was applied for the multivariable regression model. We considered six significant factors in the final multivariable model (Table 3); this requires a sample size of 30–60 cases. Our study has sufficient data for sample size calculation.

**Table 3:** Multivariable logistics model association between CIN and CV/CKD-EPI ratio.

**Variables**	**OR (95% CI)**	
	**Univariate model**	Multivariable adjusted model^*^
CV/CKD-EPI (≥1.78)	4.64 (2.84–7.56)	2.66 (1.50–4.72)
Cardiogenic shock	25.16 (11.69–54.15)	6.39 (2.53–16.12)
LVEF <40%	5.31 (3.16–8.91)	3.04 (1.67–5.54)
Min of hemoglobin	0.96 (0.95–0.97)	0.98 (0.97–0.99)
CK	1.01 (1.00–1.01)	1.01 (1.00–1.01)
Mehran integer score	1.19 (1.15–1.24)	1.08 (1.03–1.14)

* *Variables considered but not included in the final model. CHF, hypoalbuminemia, preprocedural plasma glucose, Hct, BUN, ALB, CK-MB, diseased multivessel, PCI, number of stents, emergent PCI, and exceeding MACD*.

*CI, confidence interval; CK, creatine kinase; CKD-EPI, chronic kidney disease epidemiology collaboration; LVEF, left ventricular ejection fraction; OR, odds ratio*.

## Results

### Clinical and Laboratory Characteristics of the Patients

We consecutively included a total of 4,254 patients who underwent CAG during the study period (Supplementary Figure 1). Of them, 69 patients (1.7%) suffered from CIN. Baseline clinical and angiographic characteristics, as well as the main procedural data of these patients, are listed in Table 1. The median contrast dose was 100 ml (interquartile range: 50–125 ml). The baseline characteristics of the patients with and with no CIN are shown in Table 1. The CIN patients were more likely to be elderly and had anemia, cardiogenic shock, congestive heart failure (CHF), cardiovascular disease (CVD), heart failure (HF), hypoalbuminemia, peri-hypotension, peri-intra-aortic balloon pump (peri-IABP), and LVEF below 40%. The patients with CIN were more likely to have lower laboratory measurements at min of hemoglobin (the lowest value of multiple hemoglobin check after admission), hematocrit (Hct), albumin (ALB) and higher preprocedural plasma glucose, blood urea nitrogen (BUN), creatine kinase (CK), creatine kinase-muscle/brain (CK-MB), as well as a higher CV/GFR ratio. They were more likely to have multivessel CAD and to receive PCI, stent implantation, emergent PCI, and exceeding maximum contrast dose (MACD). The median CV/CKD-EPI was 2.16 (1.30–3.93) for those with CIN and 1.15 (0.66–1.79) for those without CIN (*p* < 0.001). The median CV/C-G in patients with CIN [2.50 (1.45–4.39)] was significantly higher than those without CIN [1.27 (0.74–2.01)] (*p* < 0.001).

### CV/GFR Predicting CIN

ROC analysis demonstrated that the AUC for the CV/CKD-EPI was 0.736, and the optimal cutoff was 1.78 for CIN (Figure 1). CV/CKD-EPI was shown as the most concise equation requiring only one variable. At this cutoff value, the sensitivity and specificity were 61% and 75%, respectively. The equation CV/C-G showed a high discrimination as CV/CKD-EPI (AUC = 0.720) and showed a high reclassification as CV/CKD-EPI (IDI = 0.012, NRI = −0.162) with an optimal cutoff of 2.23 (Table 2). CV/C-G exhibited 59% sensitivity and 80% specificity for detecting CIN. However, CV/C-G showed lower calibration than CV/CKD-EPI as its higher AIC and Brier score; meanwhile, two variables are needed in the CV/C-G equation. In addition, the discrimination, calibration, and reclassification ability of CV/FAS, CV/MDRD, and CV/aMDRD were significantly lower than those of CV/CKD-EPI. CV/MDRD also need two more variables in the equation.

A non-linear association between CV/CKD-EPI and CIN was demonstrated (Figure 2). Odds of CIN were low, and linear association until the CV/CKD-EPI was 1.78, at which point a positive exponential association emerged. According to the univariate logistic regression analysis, a CV/CKD-EPI >1.78 was a significant predictor of CIN [odds ratio (OR) = 4.64, 95% CI = 2.84–7.56, *p* < 0.001) (Table 3). In the multivariable analysis, CV/CKD-EPI >1.78 (OR = 2.66, 95% CI = 1.50–4.72, *p* < 0.001) remained an independent risk factor for CIN after adjusting for other potential confounders.

On the other hand, CV/GFR on the basis of renal function performed better MACD in predicting CIN in this study (AUC = 0.736 *vs*. AUC = 0.552, *p* < 0.001).

### Impact of CV/GFR and CIN on Subgroup

When the incidence of CIN was assessed in the subsets stratified by age (<60 *vs*. ≥60), a higher incidence of CIN was evident in the CV/CKD-EPI ≥1.78(*p* < 0.001) (Figure 3). Similar trends were observed in the categories of PCI status (elective vs. acute), LVEF (<40 *vs*. ≥40), and STEMI (with vs. without). However, it was observed only in patients with no cardiogenic shock (*p* < 0.001) or high-risk level of Mehran score. We observed a significant additive interaction between CV/CKD-EPI and level of Mehran score, with interaction contrast ratio of 9.13 (95% CI = 2.19–16.07), *p* = 0.009. Notably, adjusted ORs for CV/CKD-EPI ≥1.78 in predicting CIN for the low-risk to the high-risk level of Mehran score were 3.51 (95% CI = 1.62–7.60) and 13.34 (95% CI = 6.77–26.25) (data not shown).

## Discussion

### Key Findings

The CV/CKD-EPI was a simple but high-efficiency tool for guiding contrast dosing in patients undergoing CAG or PCI. It was superior to CV/C-G, CV/FAS, CV/MDRD, and CV/aMDRD in model fit performance. The CV/CKD-EPI ≥1.78 was associated with a high incidence of CIN. Similar results were found in the subgroup analysis, especially in high-risk level of Mehran score.

### CIN Definition and Incidence

The incidence of CIN varies widely across studies, depending on the varying patient samples, different baseline risk factors, and the disparities in definitions (32). Gurm et al. defined CIN as ≥0.5 mg/dl absolute increase in Scr from baseline, but creatinine collected in the follow-up period was variable because of different lengths of hospital stay (13). Laskey et al. defined CIN as an absolute increase in serum creatinine of >0.5 mg/dl in 24–48 h. Because there was no widely accepted alternative term, our research defined the CIN term as Laskey (3).

### CV Dose

The CV administered during a cardiovascular procedure is crucial. Over the past years, the suggested volume cutoff has varied from a fixed volume of 125 ml (33) to an relative volume of MACD (4) or a relative volume of GFR. MACD was defined by an empiric formula of 5 ml of body weight (kg)/baseline Scr (mg/dl), with a maximum dose of 300 ml. It is hypothesized that contrast dose only associated with body weight and the baseline kidney function, but there was no scientific basis. Even though MACD is frequently used in clinical practices, the CIN still occurs even when MACD is not exceeded, such as the incidence of 11% found by Ogata et al. (34) and 13% concluded by Marenzi et al.

On the contrary, CV/GFR on the basis of renal function showed better performance than MACD in predicting CIN in our study, which is similar to previous studies (13). Raposeiras-Roubin et al., Nyman et al., Laskey et al., and Gurm et al. were the pioneers to propose the use of CV/GFR (3, 13, 17, 35). Raposeiras-Roubin et al. founded there were no differences in the discriminative ability to predict CIN between the three GFR equations (CV/MDRD, CV/C-G, and CV/CKD-EPI) (17) based on CAG patients with acute coronary syndrome from a retrospective cohort in Spain. Nyman et al. reported that at fixed CV/C-G ratios (from 3:1 ratio to 1:2 ratio), CIN risk increased marginally with decreasing eGFR among patients who underwent CAG for STEMI in a Swedish cohort study (35). Laskey et al. recommended a CV/C-G cutoff value >3.7 for evaluating the safe volume, but they also stated that a small but significant number of patients would develop CIN even when the ratio is <3.7. It is consistent with our finding that 17% (12/69) true-positive CIN patients with a CV/C-G value below 3.7 were misclassified to negative. Our results provided further support for the hypothesis that lower contrast media volume by CV/CKD-EPI exceeding 1.78 was an independent predictor of CIN (7).

Overall, routine measurements of the maximum limit of contrast volume in CAG or PCI, either using the MACD or CV/GFR method, are essential and should be recommended before the procedure.

### GFR Algorithm

When defining the ratio for contrast dosing, the variety of GFR formula should be taken into account, such as CV/CKD-EPI (18, 19, 21), CV/C-G (16), CV/FAS (22), CV/MDRD (23, 24), and CV/aMDRD (25), which were generally seen in clinic. It is widely accepted that CKD-EPI gives the best estimation of GFR based on a gold standard measurement using I-iothalamate (19). However, to our knowledge, these equations have not been externally validated to calculate the cutoff ratio in CAG or PCI patients together. By comparing the above five equations of contrast dosing ratio, we found that CV/CKD-EPI was the best equation for guiding reduction in the contrast nephrotoxicity.

### Strengths and Limitations

Our multicenter prospective cohort study supports the need for minimizing contrast dose in CAG or PCI procedures. The usage of CV/CKD-EPI follows the basic pharmacological principles, and our findings demonstrate a consistent relationship between the high incidence of CIN and CV/CKD-EPI in total patients and in subgroups patients. Furthermore, the inherent simplicity and convenience of calculating CV/CKD-EPI make this indicator an easy method in routine clinical practice.

This study possessed several limitations. First, the cohort included Chinese patients only, which may potentially limit the generalizability of our results to other countries and territories. On the other hand, because of the relative large population, the prospective nature, and the multicenter recruitment, our results may provide more reliable evidence than previous single-center studies (7, 9, 10, 36, 37). Second, the CV/GFR was computed using five primary formulas, rather than a direct measurement. Despite this, most of the equations have been established and validated in Western countries, and the MDRD-cn formula was the same accuracy as MDRD. Third, patients who were excluded due to absence of post-PCI serum creatinine ascertainment were, in general, healthier than those in this cohort, and this might introduce potential selection bias. However, we observed a similar relationship in patients who underwent elective PCI and had less baseline risk factors of renal complications.

## Conclusions

In conclusion, intraprocedural preventive measures should include a priori calculation of CV/GFR to limit contrast volume, and the equation of CKD-EPI showed better performance in estimating GFR than others. Future guidelines to prevent CIN should consider incorporating a more objective measurement of CV such as CV/GFR.

## Data Availability Statement

The original contributions presented in the study are included in the article/Supplementary Material, further inquiries can be directed to the corresponding author/s.

## Ethics Statement

The studies involving human participants were reviewed and approved by the institutional Ethics Research Committee of Guangdong General Hospital (No. GDREC2012141H). The patients/participants provided their written informed consent to participate in this study. The patients/participants provided their written informed consent to participate in this study.

## Author Contributions

ZN and ZL conceived and designed the research. ZN drafted the manuscript, analyzed, and interpreted the data. YL, CW, GC, and GS collected data and revised the manuscript critically for important intellectual content. All authors contributed to the article and approved the submitted version.

## Conflict of Interest

The authors declare that the research was conducted in the absence of any commercial or financial relationships that could be construed as a potential conflict of interest.

## Publisher's Note

All claims expressed in this article are solely those of the authors and do not necessarily represent those of their affiliated organizations, or those of the publisher, the editors and the reviewers. Any product that may be evaluated in this article, or claim that may be made by its manufacturer, is not guaranteed or endorsed by the publisher.
