# Supplementary material for: Safe Limits of Contrast Media for Contrast-Induced Nephropathy: A Multicenter Prospective Cohort Study
Source: Front Med (Lausanne). 2021 Aug 20;8:701062. doi: 10.3389/fmed.2021.701062 (PMC8417794; doi:10.3389/fmed.2021.701062)
Supplement: Supplementary file 1 [file Data_Sheet_1.docx]

**Supplementary Table S1** **Ten Equations of eGFR**

| eGFR definition, ml/min/1.73 m2 | Equations (Scr unit, μmol/l) | Perform population |
| --- | --- | --- |
| BIS1(36) | 3736 × (Scr/88.4)^-0.87^ × age^-0.95^ × 0.82 (if female) | Only elder adult but poor performance |
| C-G(16) | (140-age) ×weight/(Scr×0.01131×72) ×0.85 (if female) | Not suitable for child and older adults, 236 adults, predominantly male |
| CKD-EPI(18) | Male and Scr ≤80: 141 × [Scr / (0.9 × 88.4)]^–0.411^ × 0.993^age^  Male and Scr >80: 141 × [Scr / (0.9 × 88.4)]^–1.209^ × 0.993 ^age^  Female and Scr ≤62: 144 × [Scr / (0.7 × 88.4)]^–0.329^ × 0.993 ^age^  Female and Scr >62: 144 × [Scr / (0.7 × 88.4)]^–1.209^ × 0.993 ^age^ | Full age, 8254 CKD individuals |
| FAS(21) | Age≤ 40 years: 107.3/(Scr/Q)  Age> 40 years: 107.3/(Scr/Q) ×0.988^(Age – 40)^  Q = 80(male ≥20 years), Q=62(female ≥ 20 years) | Full age, 6870 individuals |
| LM-rev(37) | e^X-0.0158 × age + 0.438 × ln(age)^  Female and Scr < 150: X = 2.50 + 0.0121 × (150-Scr)  Female and Scr ≥ 150: X = 2.50 – 0.926 × ln(Scr/150)  Male and Scr < 180: X= 2.56 + 0.00968 × (180-Scr)  Male and Scr ≥ 180: X = 2.56 – 0.926 × ln(Scr/180) | Full age, 850 predominantly Swede, no external validation. |
| MDRD(22) | MDRD7: Male:170 × (Scr/88.4)^-0.999^ ×(age)^-0.176^ × (serum urea/0.357)^-0.170^ × (serum albumin/10)^0.318^ × 0.762 (if female)  serum urea, mmol/l; albumin, g/l. | Full age, 1785 CKD individuals |
| aMDRD(24) | 186 * (Scr/88.4)^-1.154^ * (age)^-0.203^ × 0.742 (if female) | Full age, 253 samples from MDRD study |
| MDRD-IDMS(24) | 175 * (standardized Scr/88.4)^-1.154^ * (age)^-0.203^ × 0.742 (if female) | Full age, 253 samples from MDRD study, standardized Scr traceable to isotope dilution mass spectrometry at NIST |
| MDRD-cn(23) | MDRD7-cn: MDRD7× 1.211  aMDRD-cn: a-MDRD× 1.233 | Full age, 684 CKD individuals, Chinese |

To convert GFR from ml/min per 1.73 m2 to ml/s per 1.73 m2, multiply by 0.0167. To convert Scr from μmol/l to mg/dl, divide by 88.4.

Abbreviations: a-MDRD, abbreviated Modification of Diet in Renal Disease; BIS1 = Berlin Initiative Study 1;C-G, Cockcroft–Gault; CKD-EPI, Chronic Kidney Disease Epidemiology Collaboration; eGFR, estimating glomerular filtration rate; FAS, Full Age Spectrum; LM-rev, revised Lund-Malmö; MDRD, Modification of Diet in Renal Disease Study; MDRD -IDMS, Modification of Diet in Renal Disease Study values based on isotope dilution mass spectrometry; Scr, serum creatinine.

**Supplementary Table S2 Investigator or Sub-Investigator/RESCIND Groups and Institution List**

| No. | Investigator or sub-investigator | Institution |
| --- | --- | --- |
| 01 | Jiyan Chen, Ning Tan, Yingling Zhou, Yong Liu, Shiqun Chen, Jianfang Luo, Danqing Yu, Liwen Li, Zhujun Chen, Guang Li, Bin Zhang, Lijun Jin, Hong Yan, Taiming Dong Yuan Liu, Wenhui Huang, Junqing Yang, Pengcheng He, Nianjin Xie. | Guangdong provincial Key Laboratory of Coronary Heart Disease Prevention,  Guangdong Cardiovascular institute, Guangdong Academy of Medical Sciences, Guangdong General Hospital |
| 02 | Zhimin Du, Yi Li, Ming Long | The First Affiliated Hospital, Sun Yat-sen University |
| 03 | Jingfeng Wang, Ruqiong Nie | Sun Yat-sen Memorial Hospital, Sun Yat-sen University |
| 04 | Yuqing Hou, Jiancheng Xiu, Zheng Huang | Nanfang Hospital, South medical university |
| 05 | Jian Qiu, Dingcheng Xian, Changjiang Hong | General Hospital of Guangzhou Military Command of People’s Liberation Army |
| 06 | Ken Wu, Chang Peng | Affiliated Hospital of Guangdong Medical University |
| 07 | Guoliang Jia, Qibing Cai | Dongguan Kanghua Hospital |
| 08 | Jianfeng Ye, Shaohui Su | Dongguan People's Hospital |
| 09 | Yan Liang, Cong Chen | Maoming People's Hospital |
| 10 | Guifu Wu, Jiansheng Wu | The Fourth People's Hospital Shenzhen |
| 11 | Kaihong Chen, Liling Chen | Longyan First Hospital |
| 12 | Xiaoguang Zhou, Yuying Hu, Haiyan Wei | First People's Hospital of Kashgar |

**Supplementary Table S3** **Baseline Characteristics of Patients in the REICIN Study, Stratified by Variables That Were Available for Imputation**

| **Characteristic** | **Number of cases with  missing information** | **Complete cases (n = 2,574)** | **Patients with at least  one missing value (n = 1,679)** | **P-value**† |
| --- | --- | --- | --- | --- |
| Age, years | 0 (0) | 63 (58–72) | 63 (56–72) | 0.082 |
| Male | 0 (0) | 1,943 (75.0) | 1,224 (72.9) | 0.133 |
| Weight, kg | 358 (8.3) | 65.0 (58.0–72.5) | 65.0 (59.0–69.0) | 0.001 |
| BMI | 684 (16.0) | 24.0 (21.9–26.1) | 24.0 (21.7–25.8) | <0.001 |
| Systolic BP, mmHg | 0 (0) | 130 (118–143) | 130 (1119–146) | 0.120 |
| Diastolic BP, mmHg | 0 (0) | 75 (68–83) | 76 (69–84) | 0.033 |
| HR, bpm | 0 (0) | 74 (67–82) | 75 (67–82) | 0.616 |
| **Medical history** |  |  |  |  |
| Diabetes mellitus | 0 (0) | 741 (28.6) | 422 (25.1) | 0.013 |
| Hypertension | 0 (0) | 1,440 (55.6) | 910 (54.2) | 0.384 |
| Previous CABG | 0 (0) | 5 (0.2) | 2 (0.1) | 0.560 |
| Hyperlipidemia | 0 (0) | 325 (12.5) | 192 (11.4) | 0.280 |
| Anemia | 0 (0) | 755 (12.5) | 495 (29.5) | 0.280 |
| History of smoking | 0 (0) | 1,043 (40.2) | 551 (32.8) | <0.001 |
| Previous MI | 0 (0) | 257 (9.9) | 139 (8.3) | 0.074 |
| PVD | 0 (0) | 3 (0.1) | 3 (0.2) | 0.592 |
| Anterior infarction | 0 (0) | 235 (9.1) | 157 (9.4) | 0.953 |
| Cardiogenic shock | 0 (0) | 15 (0.6) | 24 (1.4) | 0.004 |
| CHF | 0 (0) | 502 (19.4) | 448 (26.7) | <0.001 |
| CVD | 0 (0) | 104 (4.0) | 79 (4.7) | 0.275 |
| HF | 0 (0) | 429 (16.6) | 411 (24.5) | <0.001 |
| Hypoalbuminemia | 0 (0) | 113 (4.4) | 90 (5.4) | 0.133 |
| Stroke | 0 (0) | 104 (4.0) | 79 (4.7) | 0.275 |
| **Clinical conditions** |  |  |  |  |
| Presence of ACS | 0 (0) | 1,247 (48.1) | 750 (44.7) | 0.089 |
| UA/NSTEMI | 0 (0) | 1092 (42.1) | 681 (40.6) | 0.596 |
| Anterior STEMI | 0 (0) | 609 (23.5) | 396 (23.6) | 0.998 |
| Cardiac arrest | 0 (0) | 6 (0.2) | 1 (0.1) | 0.175 |
| Peri-hypotension | 0 (0) | 43 (1.7) | 82 (4.9) | <0.001 |
| Peri-IABP | 0 (0) | 32 (1.2) | 49 (2.9) | <0.001 |
| LVEF | 544 (12.7) | 62 (55–67) | 60.0 (51.7–66.0) | <0.001 |
| NYHA class level | 0 (0) |  |  | 0.889 |
| ≥3 |  | 194 (7.5) | 140 (8.3) |  |
| 2 |  | 847 (32.7) | 536 (31.9) |  |
| 1 |  | 1,551 (59.8) | 1,003 (59.7) |  |
| Killip class level | 0 (0) |  |  | <0.001 |
| ≥3 |  | 78(3.0) | 104(6.2) |  |
| 2 |  | 257(9.9) | 174 (10.4) |  |
| 1 |  | 2,257 (87.1) | 1,401 (83.4) |  |
| **Laboratory measurements** |  |  |  |  |
| Pre-procedural plasma glucose | 0 (0) | 6.1 (5.1–8.1) | 6.1 (5.1–8.1) | 0.220 |
| Min of hemoglobin | 55 (1.3) | 132.0 (120.0–142.5) | 131.8 (119.3–143.0) | 0.636 |
| Hct | 49 (1.1) | 40.3 (37.1–43.2) | 40.4 (37.1–43.1) | 0.340 |
| Scr, mg/dl | 0 (0) | 85 (72–102) | 82 (69–100) | 0.001 |
| Creatinine clearance, ng/ml | 0 (0) | 70.7 (52.7–88.7) | 70.3 (52.6–88.7) | 0.845 |
| eGFR, ml/min/1.73mm^2^ | 0 (0) | 80.5 (65.0–95.2) | 83.1 (64.7–98.5) | 0.004 |
| BUN | 37 (0.9) | 5.1 (4.1–6.4) | 5.1 (4.1–6.4) | 0.961 |
| HDL-C, mmol/L | 129 (3) | 1.0 (0.8–1.1) | 1.0 (0.9–1.2) | <0.001 |
| ALB, mmol/L | 251 (5.9) | 37.1 (34.5–39.5) | 37.5 (34.6–40.1) | 0.003 |
| CK | 210 (4.9) | 93.0 (64.0–152.0) | 97.1 (75.0–175.0) | <0.001 |
| CK-MB | 423 (9.9) | 10.3 (6.9–15.7) | 14.2 (7.2–55.4) | <0.001 |
| **Medication** |  |  |  |  |
| Pre-ACEI/ARB | 0 (0) | 604 (23.3) | 369 (22.0) | 0.313 |
| Pre-β block | 0 (0) | 1,808 (69.8) | 885 (52.7) | <0.001 |
| Pre-CCB | 0 (0) | 460 (17.7) | 287 (17.1) | 0.5836 |
| Pre-statins | 0 (0) | 2,311 (89.2) | 1,397 (83.2) | <0.001 |
| Pre-antidiabetics | 0 (0) | 475 (18.3) | 229 (13.6) | <0.001 |
| Pre-metformin | 0 (0) | 84 (3.2) | 40 (2.4) | 0.103 |
| Pre-diuretics | 0 (0) | 454 (17.5) | 289 (17.2) | 0.799 |
| **Procedure** |  |  |  |  |
| Multivessel stent | 0 (0) | 416 (16.0) | 260 (15.5) | 0.622 |
| Diseased vessel≥1 | 0 (0) | 2,107 (81.3) | 1,330 (79.2) | 0.095 |
| Diseased multivessel | 0 (0) | 1528 (59.3) | 916 (54.6) | 0.005 |
| CTO | 0 (0) | 484 (18.7) | 250 (14.9) | 0.001 |
| PCI | 0 (0) | 1,596 (61.6) | 981 (58.4) | 0.040 |
| Number of stents | 0 (0) | 1.0 (0.0–2.0) | 1.0 (0.0–2.0) | 0.237 |
| Emergent PCI | 0 (0) | 315 (12.2) | 194 (11.6) | 0.841 |
| **Contrast type** |  |  |  |  |
| Iopamidol, [iso-osmia](http://dict.youdao.com/w/iso-osmia/#keyfrom=E2Ctranslation) | 0 (0) | 340 (13.1) | 281 (16.7) | 0.001 |
| Contrast volume, ml | 0 (0) | 100 (50–130) | 100 (50–120) | 0.217 |
| **Mehran score** |  |  |  |  |
| Mehran integer score | 0 (0) | 4.0 (1.0–6.6) | 4.0 (1.0–7.7) | 0.009 |
| Mehran integer score level | 0 (0) |  |  | <0.001 |
| Very high (≥16) |  | 32 (1.2) | 63 (3.8) |  |
| High (11-16) |  | 211 (8.1) | 183 (10.9) |  |
| Medium (6–10), |  | 651 (25.1) | 421 (25.1) |  |
| Low (≤5) |  | 1,698 (65.6) | 1,012 (60.2) |  |

Categorical data are presented as number (%), continuous data are presented as median (interquartile range).

Notes: *Data includes imputed data for those with missing values.

Abbreviations: ACEI/ARB, angiotensin converting enzyme inhibitor/angiotensin receptor blocker; ACS, acute coronary syndrome; ALB, albumin; ARF, acute renal failure; BMI, body mass index; BP, blood pressure; BUN, blood urea nitrogen; CABG, coronary artery bypass graft; CCB, calcium channel blocker; CHF, congestive heart failure; CK, creatine kinase; CK-MB, creatine kinase-muscle/brain; CTO, chronic total occlusion; CVD, cardiovascular disease; eGFR, estimated glomerular filtration rate; Hct, hematocrit; HDL-C, high-density lipoprotein cholesterol; HF, heart failure; HR, heart rate; IABP, intra-aortic balloon pump; LVEF, left ventricular ejection fraction; MI, acute myocardial infarction; NSTEMI, non-ST-elevation myocardial infarction; NYHA, New York Heart Association; PCI, percutaneous coronary intervention; PVD, peripheral vascular disease; Scr, serum creatinine; UA, unstable angina.
