# Supplementary figures and images for: Safe Limits of Contrast Media for Contrast-Induced Nephropathy: A Multicenter Prospective Cohort Study
Source: Front Med (Lausanne). 2021 Aug 20;8:701062. doi: 10.3389/fmed.2021.701062 (PMC8417794; doi:10.3389/fmed.2021.701062)

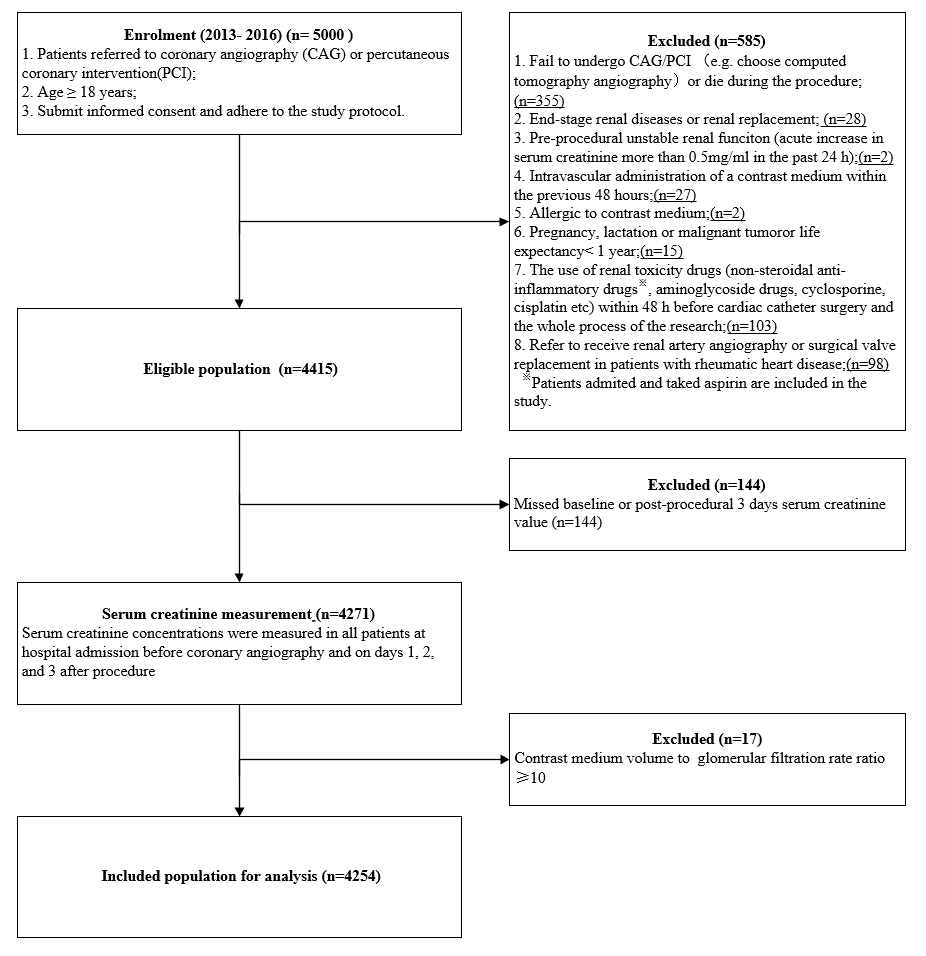

Supplement: Supplementary file 2 [file Image_1.TIF]
